# Biomechanical analysis of ponte osteotomy and PSO osteotomy in the treatment of ankylosing spondylitis with thoracolumbar kyphotic deformity

**DOI:** 10.3389/fsurg.2025.1730301

**Published:** 2026-01-06

**Authors:** Xu Zhu, Alimujiang Yusufu, Ajiguli Waisiding, Yuan Ma

**Affiliations:** Department of Spinal Surgery, Sixth Affiliated Hospital of Xinjiang Medical University, Ürümqi, China

**Keywords:** ankylosing spondylitis, osteotomy, ponte osteotomy, PSO osteotomy, biomechanics

## Abstract

**Background:**

Ankylosing spondylitis (AS) commonly progresses to thoracolumbar kyphosis. Pedicle subtraction osteotomy (PSO) and Ponte osteotomy are frequently used surgical methods. However, systematic comparative biomechanical studies of these two methods are insufficient, and differences in postoperative mechanical complication risks remain unclear.

**Objective:**

To compare the biomechanical characteristics of Ponte and PSO osteotomies in the treatment of AS with thoracolumbar kyphotic deformity, providing a biomechanical basis for clinical surgical decision-making.

**Methods:**

Finite element models representing unresected (M0), Ponte osteotomy (M1), and PSO osteotomy (M2) conditions were constructed based on CT data from an AS patient. A vertical load of 500 N and moments of 10 N·m in each direction were applied to the T3 vertebral body. Six loading conditions, including flexion and extension, were simulated. Spinal range of motion (ROM), vertebral stress, internal fixation stress, and displacement were analyzed.

**Results:**

Model validation showed that ROM of M0 was consistent with previous studies. After surgery, ROM significantly decreased in both osteotomies compared with M0, with M2 showing lower ROM than M1. In the M1 model, stress in the T3–T5 vertebral bodies decreased, but stress in T6 did not significantly change. In the M2 model, stress in T4–T5 vertebral bodies decreased, while stress in T7–T8 increased. Internal fixation stress in M1 was significantly lower than in M2 across all loading conditions, although displacement was greater in M1.

**Conclusion:**

Ponte osteotomy distributes stress across multiple segments, reducing internal fixation load, and is therefore suitable for moderate kyphotic deformities. PSO osteotomy provides superior corrective capability but concentrates stress on distal vertebral segments, making it suitable for severe deformities. These results can guide clinical decisions for individualized surgical selection.

## Introduction

Ankylosing spondylitis (AS) is a chronic inflammatory disease characterized by gradual ossification of spinal ligaments, fibrous rings, and intervertebral joints as the disease progresses (1, 2). With the progressive development of thoracolumbar kyphotic deformity, compensatory backward rotation of the pelvis occurs to maintain overall sagittal balance. In advanced stages, symptoms such as low back pain, difficulty maintaining upright posture, and inability to gaze forward may develop (1, 3).

Pedicle subtraction osteotomy (PSO) is widely used to treat thoracolumbar kyphotic deformities in AS. This surgical method effectively reconstructs spinal sagittal alignment and significantly improves patients' quality of life (4–6). However, previous studies have indicated that PSO is associated with postoperative mechanical complications such as rod fracture, pseudarthrosis, and proximal junctional kyphosis (7, 8). Ponte osteotomy, first described by Ponte in 1987, involves complete resection of the thoracic articular processes, partial laminae, and entire ligamentum flavum. It is used to correct thoracic kyphotic deformities, achieving an average correction of approximately 30° (9).

Although both surgical techniques are widely applied in clinical practice, systematic comparative studies regarding their biomechanical characteristics remain limited. Previous research has primarily concentrated on clinical efficacy and imaging outcomes, insufficiently addressing critical aspects such as stress distribution at osteotomy sites, biomechanical responses of adjacent segments, and internal fixation load alterations. Clarifying biomechanical differences between these two osteotomies during deformity correction is crucial for optimizing surgical decision-making and reducing postoperative internal fixation failure and adjacent segment degeneration risks. Therefore, this study aims to compare the biomechanical characteristics of Ponte and PSO osteotomies in treating AS with thoracolumbar kyphotic deformity. Biomechanical analysis was conducted to investigate spinal force lines, stress distribution at osteotomy segments, and stability of internal fixation systems. This provides a biomechanical foundation for clinical selection, aiming to enhance surgical safety and long-term outcomes.

## Materials and methods

### Research subject

The CT images were obtained from a patient diagnosed with AS complicated by thoracolumbar kyphotic deformity. The patient was a 31-year-old woman, 153 cm tall, and weighed 41 kg. The primary manifestation was significant kyphosis in the thoracolumbar region. The patient's CT data were provided by the Sixth Affiliated Hospital of Xinjiang Medical University, and the patient's family members were informed about the study protocol. This research was approved by the Medical Ethics Committee of the Sixth Affiliated Hospital of Xinjiang Medical University (Approval No.: LFYLLSC20230829-10) and complied with relevant ethical guidelines. The experiments were conducted at the Sixth Affiliated Hospital of Xinjiang Medical University from May to July 2025.

### Basic modeling procedure

#### Bone extraction and filling

**Figure 1:**
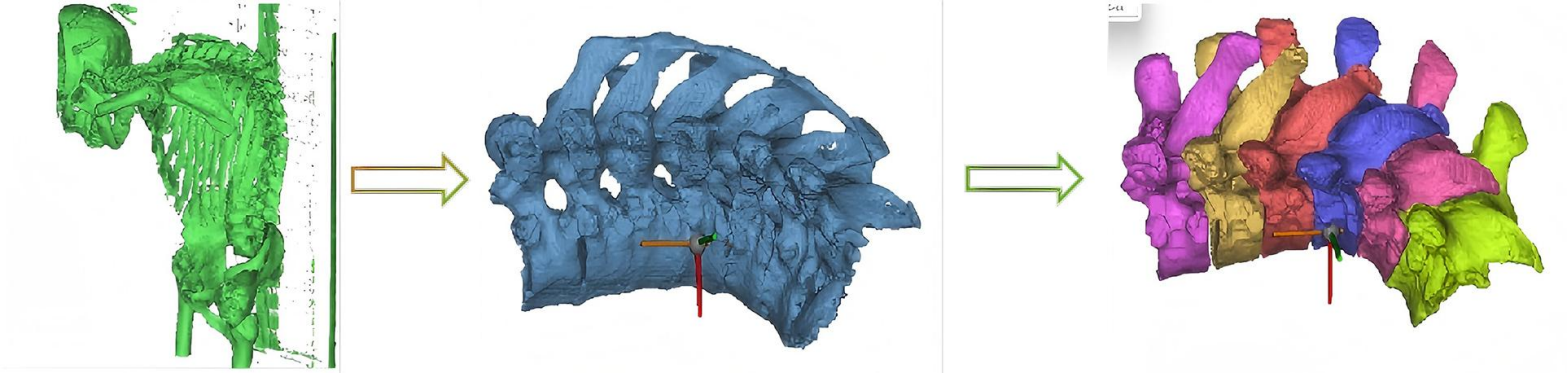
Extraction of aS thoracolumbar kyphotic bone model.

Mimics software was used to differentiate soft tissue and bone structures based on their grayscale values. Initially, the “New Mask” function and adjustments of HU value ranges were used to create masks, preliminarily separating tissues. Subsequently, the “Split Mask” function was used to isolate the target bone masks. Due to limitations in CT image quality, software recognition, and HU value settings, the “Edit Mask” function was manually applied to fill or remove gaps and redundant structures layer-by-layer on the corresponding Dicom images. After processing, masks were converted into solid models through the “Calculate Part” function and exported as STL files for further processing (shown in Figure 1).

#### Model optimization

**Figure 2:**
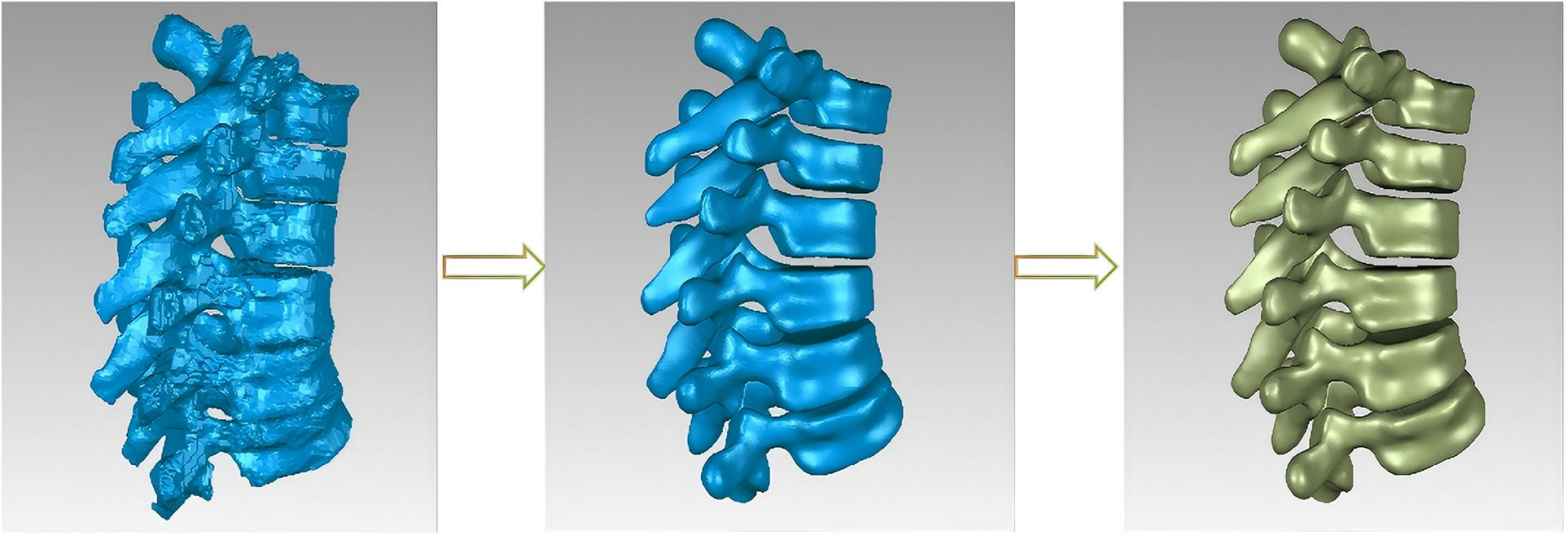
Model optimization.

(1) Shape optimization: As finite element analysis (FEA) demands high-quality geometric models, further optimization was necessary: ① The STL file generated by Mimics was imported into Geomagic software. ② The polygon module was employed for “re-meshing” to ensure mesh uniformity. ③ Surface smoothing processes including “relaxation”, “noise removal,” and “spike removal” were applied to maintain anatomical features and smooth the model surface. ④ Manual refinements were performed using the “Remove Features” function. ③ After confirming no errors using the “Mesh Doctor,” the model was duplicated and offset to generate the cancellous bone structure. (2) Surface fitting and solid model creation: After optimization, the model underwent precise surface reconstruction in Geomagic: ① “Contour exploration” identified sharp corners and irregular features. ② “Build surface patches” covered the outer surface. ③ “Repair surface patches” was used to align closely with anatomical shapes. ④ “Create grids” wrapped the surface patches; ④ “Fit surface” converted the patches into a solid model, exported in STEP format (shown in Figure 2).

#### Assembly of various models

**Figure 3:**
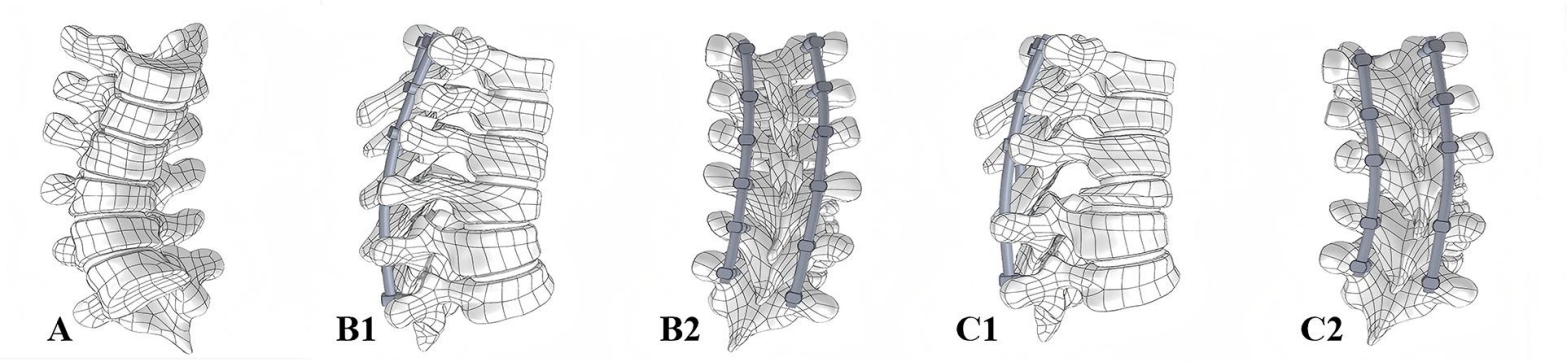
Assembly of various models. **(A)** AS model without osteotomy; **(B1/2)** AS model with Ponte osteotomy; **(C1/2**) AS model with PSO osteotomy.

The model generated in Geomagic Studio was imported into SoildWorks 2023 (Dassault Systems, USA) in IGS (Initial Graphics Exchange Specification) format. Intervertebral discs, nucleus pulposus, and articular cartilage were created, and different osteotomy procedures were simulated. The optimized models were accurately aligned and imported into Hypermesh 2021 (Altair, USA) to construct cortical bone, cancellous bone, and ligaments. Fine mesh division was carried out using appropriate meshing parameters and algorithms. These meshes served as the basis for subsequent simulation analyses, ensuring accurate and reliable results. Ansys software (Ansys, USA) was utilized for biomechanical computations (shown in Figure 3).

Mesh Generation and Material Properties The spinal model consisted of vertebral cortical bone, vertebral cancellous bone, endplates, nucleus pulposus, annulus fibrosus, articular cartilage, titanium alloy internal fixation devices, and seven related ligaments. The ligaments included supraspinous, interspinous, intertransverse, ligamentum flavum, capsular, posterior longitudinal, and anterior longitudinal ligaments. Vertebral cortical bone was simulated using triangular linear strain shell elements with a thickness of 1 mm. Vertebral cancellous bone was modeled using tetrahedral first-order linear solid elements. The endplates were represented by rectangular linear strain shell elements. The annulus fibrosus matrix, nucleus pulposus, and facet joints were modeled using eight-node hexahedral solid elements. Ligaments were considered as linear elastic, isotropic materials, modeled using one-dimensional linear rod elements. Material properties for each spinal component were defined based on references (10–12). Specific parameters are listed in Tables 1, 2.

**Table 1:** Material parameters.

Material	Young's modulus (MPa)	Poisson's ratio
Cortical bone	12,000	0.3
Cancellous bone	100	0.2
Endplate	12,000	0.3
Annulus fibrosus	12,000	0.3
Nucleus pulposus	1.0	0.49
Arthrodial cartilage	12,000	0.3
Internal fixation	110,000	0.3

**Table 2:** Material parameters (spinal ligaments).

Spring ligament	Rigidity (*N*/mm)
Lacertus medius	8.74
Ligamenta longitudinale posterius	5.83
Ligamenta interspinalia	10.85
Ligamenta supraspinale	2.39
Ligamenta intertransversaria	0.19
Ligamentum flavum	15.38
Articular capsule ligaments	15.75

For finite element construction of the AS spinal model, vertebral cancellous bone was simulated using four-node tetrahedral elements, whereas cortical bone was simulated using triangular and quadrilateral elements. The M0 model included 714,087 nodes and 437,126 elements. The M1 model contained 1,101,114 nodes and 650,708 elements. The M2 model comprised 1,002,010 nodes and 605,505 elements (Table 3).

**Table 3:** Number of elements and nodes in the finite element models.

Index	M0	M1	M2
Node	714,087	11,01,114	10,02,010
Unit	4,37,126	6,50,708	6,05,505

#### Loading conditions and observations

**Figure 4:**
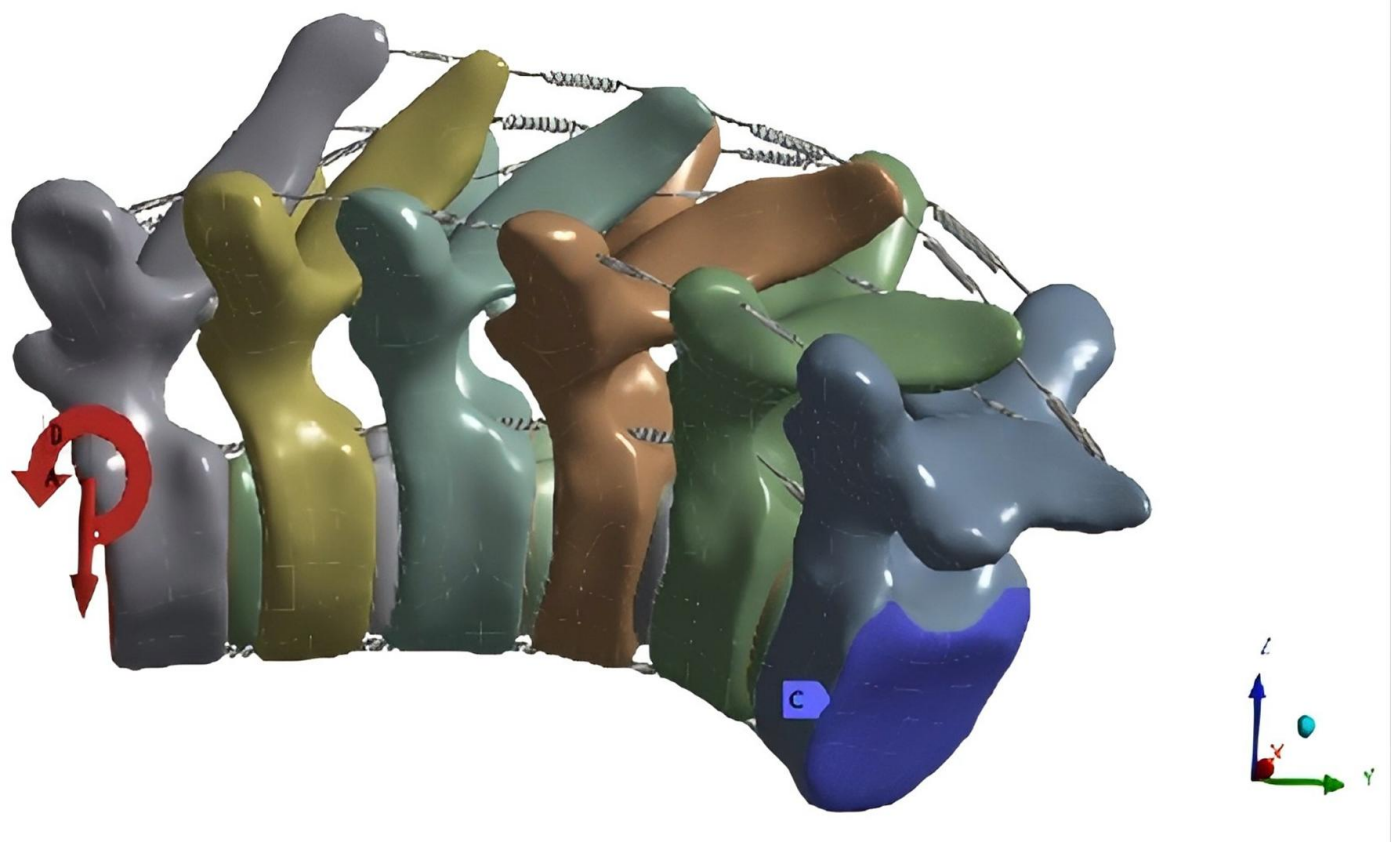
Loading diagram under forward flexion.

In the neutral position, the base of the T8 vertebral body was fixed and constrained. The upper surface of the T3 endplate was left unconstrained as the site for applied loads and torque. The “component method” was employed to determine the magnitude and direction of loading. This method simulated stress distributions due to body weight under different working conditions, ensuring loads were evenly transmitted to the surface nodes. A vertical load of 500 N and moments of 10 N·m in each direction were applied to the upper surface of the T3 lamina. This simulated forward flexion, extension, left and right lateral bending, and left and right rotation (Figure 4).

#### Main outcome measures

M0 model: Overall stress and ROM; M1 model: Stress at vertebral levels T3–T6, internal fixation stress, overall displacement, and ROM; M2 model: Stress at vertebral levels T4–T8, internal fixation stress, overall displacement, and ROM.

## Results

### Model validation

**Figure 5:**
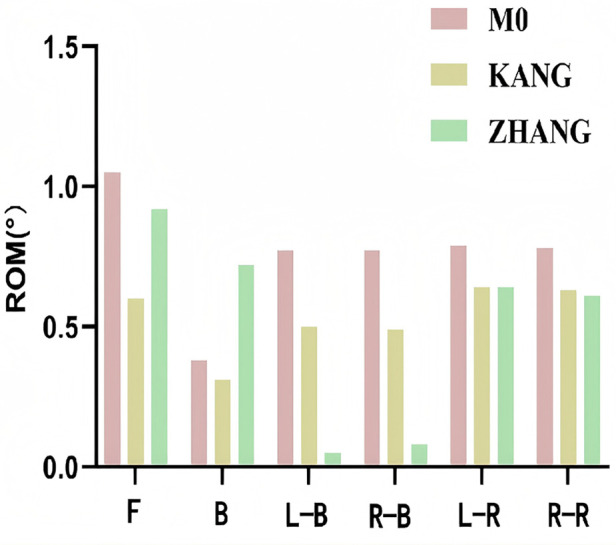
Comparison of vertebral ROM between model M0 and previous studies. F, forward flexion; B, backward extension; L-R, left rotation; R-R, right rotation; L-B, left bending; R-B, right bending.

After establishing the T3–T8 finite element model, its validity was verified. Validation is essential to ensure the accuracy of material properties and reliability of the entire spinal model. The lower surface of T8 was fixed, and a vertical axial load of 500 N and a torque of 10 N·m in all directions were applied to the upper surface of T3. These conditions simulated spinal motion under six activities. Comparing the biomechanical results with those of previous studies by Kang et al. (13) and Zhang et al. (14), the ROM of the AS model (M0) under flexion, extension, left/right bending, and left/right rotation closely matched previous findings (Table 4, Figure 5).

**Table 4:** Comparison of vertebral ROM between model M0 and previous studies.

Authors	Forward flexion	Backward extension	Left bending	Right bending	Left rotation	Right rotation
M0	1.05	0.38	0.77	0.77	0.79	0.78
Kang (12)	0.60	0.31	0.50	0.49	0.64	0.63
Zhang (13)	0.92	0.72	0.05	0.08	0.64	0.61

### Comparison of overall ROM among different models

Compared with the normal model (M0), the overall ROM of models M1 and M2 significantly decreased under all six working conditions (Table 5).

**Table 5:** Comparison of T3–T8 segment ROM in different models under various conditions (°).

Group	Forward flexion	Backward extension	Left bending	Right bending	Left rotation	Right rotation
M0	1.047	0.377	0.770	0.765	0.787	0.775
M1	0.624	0.235	0.518	0.461	0.637	0.412
M2	0.534	0.212	0.458	0.402	0.525	0.335

### Comparison of Von Mises stress extremes in T3–T6 vertebrae in model M1

**Figure 6:**
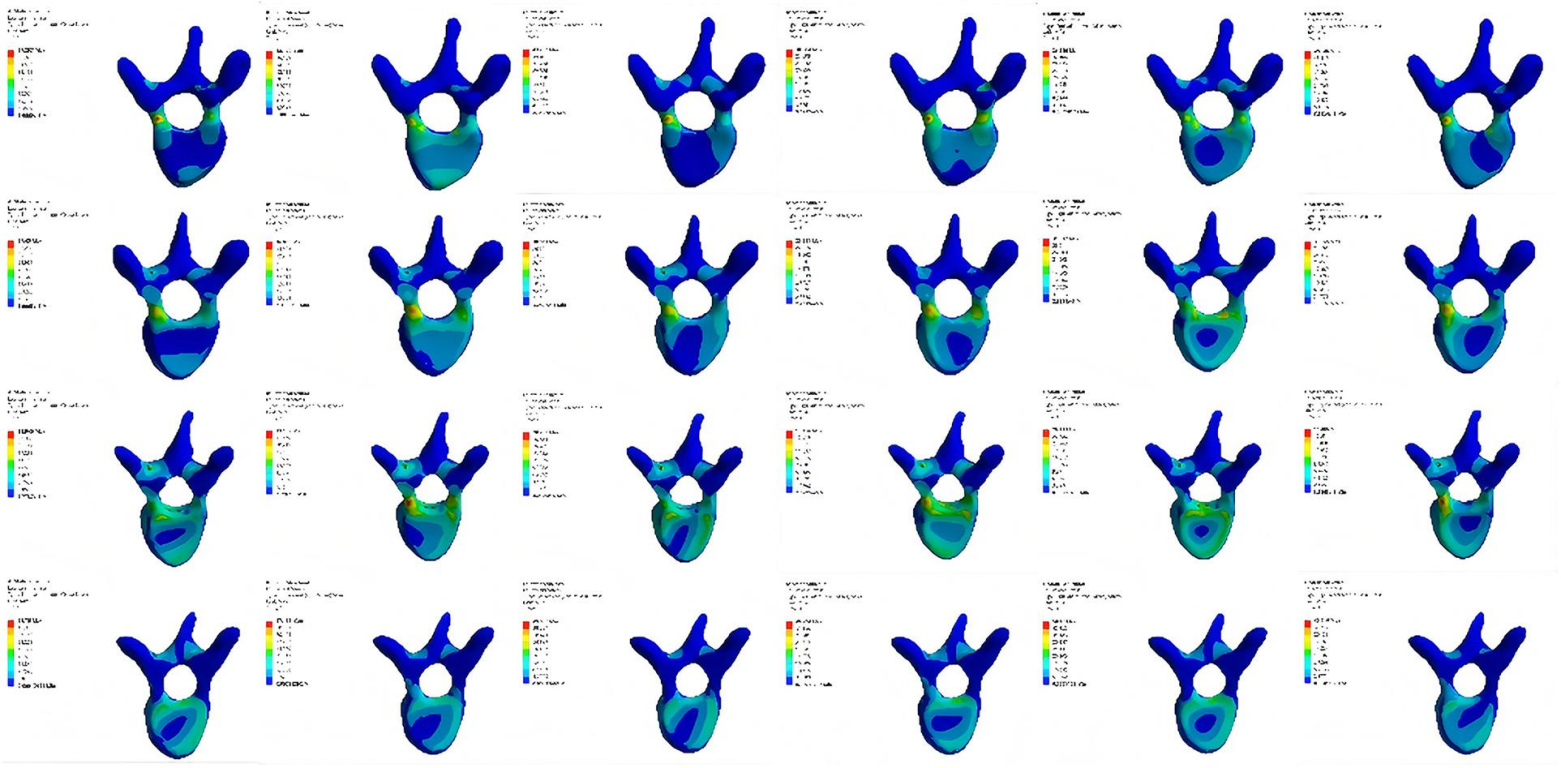
Von mises stress nephograms of T3-T6 vertebrae under different conditions in model M1. F, forward flexion; B, backward extension; L-R, left rotation; R-R, right rotation; L-B, left bending; R-B, right bending.

Compared with M0, the Von Mises stress extremes of vertebrae T3, T4, and T5 in M1 were significantly reduced. In contrast, the stress extremes of vertebra T6 in M1 remained similar to M0 under different conditions (Table 6, Figure 6).

**Table 6:** Von mises stress extremes of vertebrae under different conditions (Mpa).

Group		Forward flexion	Backward extension	Left bending	Right bending	Left rotation	Right rotation
M1	T3	57.237	18.179	43.989	30.384	36.119	48.846
	T4	37.45	16.653	30.061	25.511	31.512	31.265
	T5	25.946	13.139	20.908	17.456	29.91	19.406
	T6	58.76	37.117	49.632	49.294	50.48	45.416
M2	T4	25.881	15.889	32.33	35.622	31.461	22.031
	T5	28.371	15.33	24.716	24.897	31.96	19.898
	T7	82.603	37.254	67.718	52.283	62.257	58.235
	T8	64.276	38.496	51.203	52.336	50.351	52.757

### Comparison of Von Mises stress extremes in T4–T8 vertebrae in model M2

**Figure 7:**
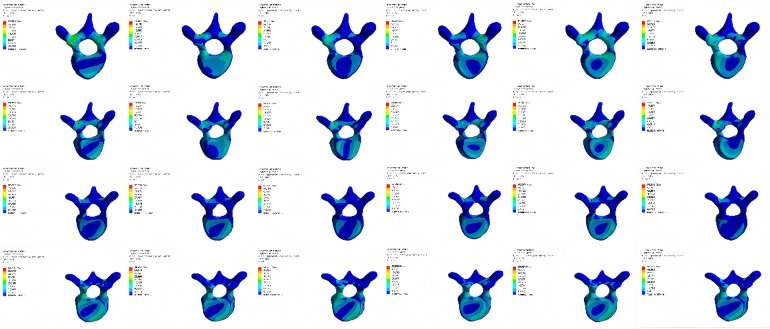
Von mises stress nephograms of T4–T8 vertebrae under different conditions in model M2.

Compared with M0, the Von Mises stress extremes of vertebrae T4 and T5 in M2 significantly decreased. However, stress extremes of vertebrae T7 and T8 notably increased and were higher than those in model M0 (Table 6, Figure 7).

### Comparison of internal fixation Von Mises stress extremes between models

**Figure 8:**
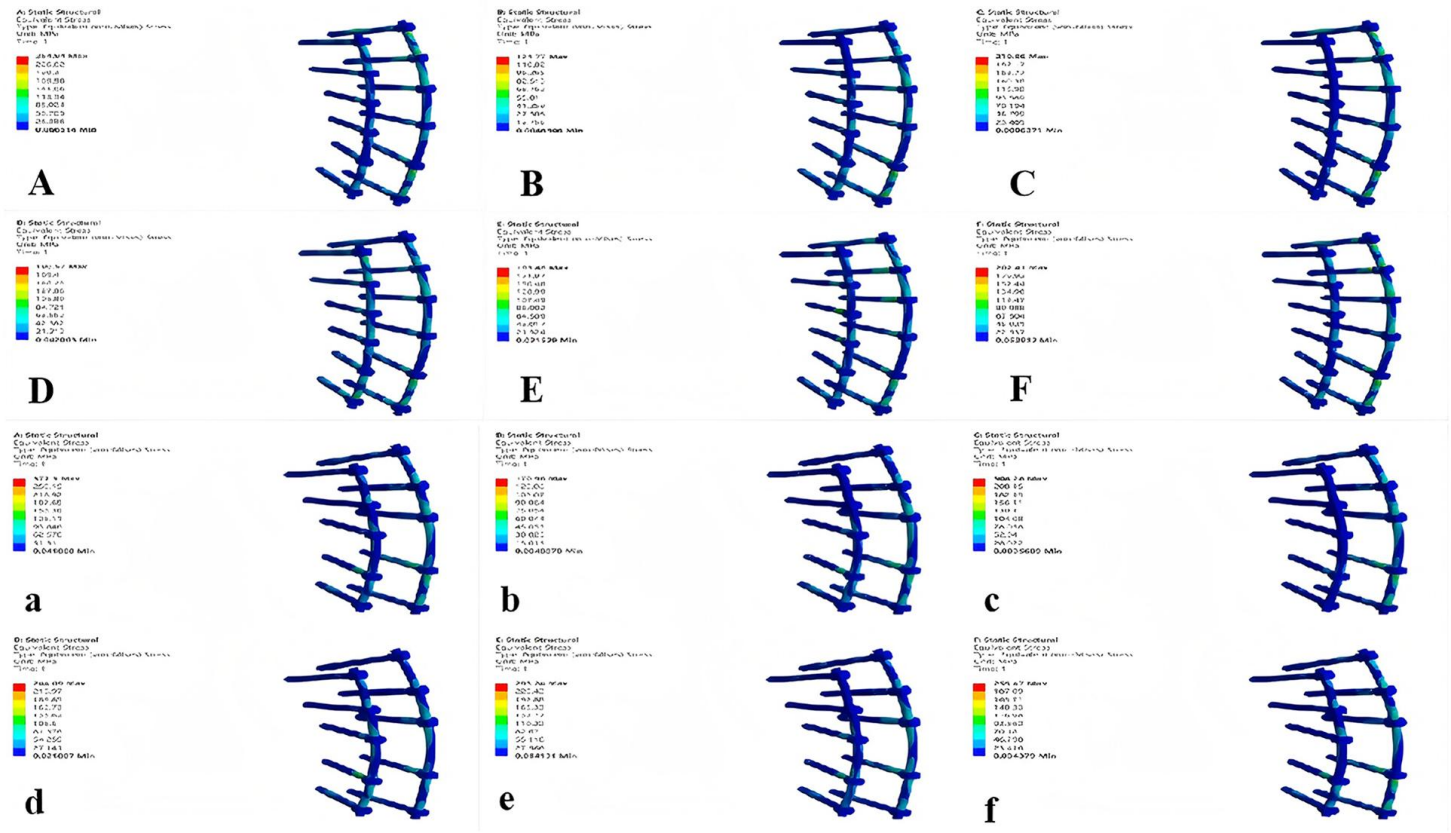
Von mises stress nephograms of internal fixation under different conditions in the two models. **(A–F)** represents models M1 and M2 under the following six conditions: flexion, extension, left/right bending, and left/right rotation.

Although model M1 exhibited greater displacement, its internal fixation stress was significantly lower than that of M2 under all loading conditions.(forward flexion, extension, left/right bending, and left/right rotation). See Table 7 and Figure 8 for details.

**Table 7:** Internal fixation stress and displacement extremes of the two models under different conditions.

Group	Forward flexion	Backward extension	Left bending	Right bending	Left rotation	Right rotation
Stress (MPa) M1	254.94	123.77	210.56	190.57	193.46	202.41
M2	377.3	170.96	304.28	244.09	293.24	255.47
Displacement M1	0.77374	0.43457	0.66745	0.58603	0.77302	0.51565
(mm) M2	0.62346	0.33917	0.52402	0.47056	0.61862	0.38819

### Comparison of internal fixation displacement extremes between models

**Figure 9:**
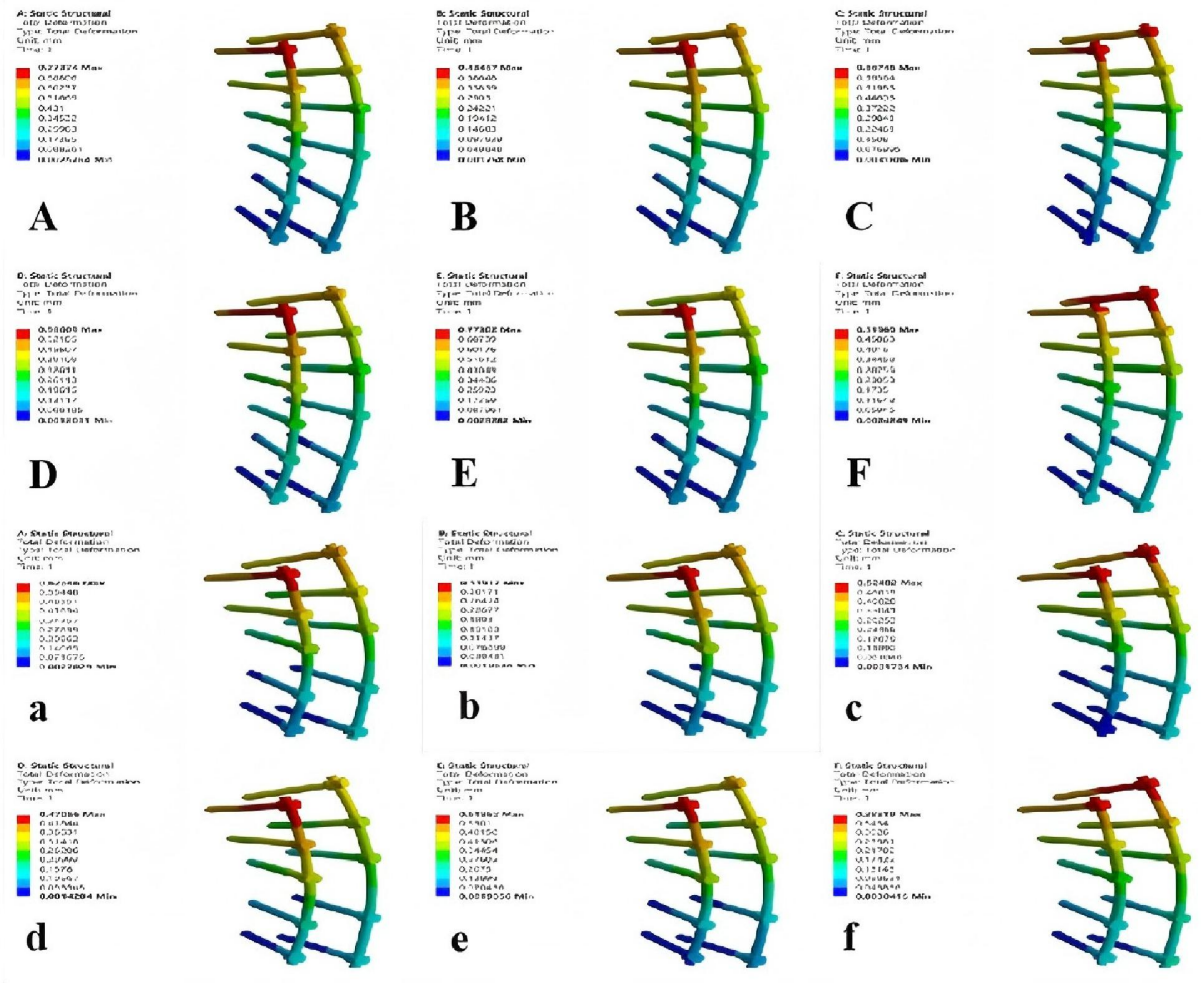
Internal fixation displacement nephograms of the two models under different conditions. **(A–F)** represents models M1 and M2 under the following six conditions: flexion, extension, left/right bending, and left/right rotation.

The internal fixation displacement extremes were significantly greater in model M1 compared to model M2 under all six working conditions (forward flexion, extension, left/right bend, and left/right rotation). See Table 7 and Figure 9 for details.

## Discussion

AS is a chronic rheumatic autoimmune disease primarily affecting the sacroiliac joints, spine, and peripheral joints. It significantly reduces the patient's quality of life and daily functioning (15, 16). Early AS symptoms typically include dull lower back pain and morning stiffness, which can improve after exercise. As the disease progresses, severe cases may involve ligament ossification, muscle stiffness, muscular atrophy, spinal rigidity, and kyphosis (17). Thoracolumbar kyphotic deformity in AS can result from minor trauma, characterized by spinal sagittal imbalance. Clinical manifestations include muscle dysfunction, fatigue, pain, and difficulty lying flat or walking normally. Thus, effective treatment of AS-related thoracolumbar kyphosis has become an important challenge in orthopedic surgery. Spinal osteotomy can effectively reconstruct sagittal spinal balance, restore horizontal gaze, and relieve organ compression in AS patients (18, 19). Surgeons may select osteotomy techniques according to the specific characteristics of spinal kyphosis, location of cervical vertebral curvature, degree of anterior longitudinal ligament calcification (bone bridge formation), and kyphotic angle magnitude (20). PSO is a progressive surgical method. It can preserve basic middle-column closure and anterior-column closure, or it can be modified into an anterior-column open osteotomy to achieve greater kyphosis correction. Therefore, PSO is widely applied in AS correction (21). Compared with higher-level osteotomies, Ponte osteotomy has the advantages of simpler operation, fewer osteotomy sites, and shorter operation time (9). However, limited reports exist on the biomechanical efficacy of these two osteotomies in treating AS with thoracolumbar kyphosis. Thus, this study established a finite element model of the human T3–T8 spine. PSO and Ponte osteotomies were simulated to analyze their biomechanical stability and provide a biomechanical basis for clinical decision-making.

Wang et al. (22) conducted a finite element analysis of titanium rods following surgical correction of Lenke type 5 adolescent idiopathic scoliosis (AIS) from T3 to L5. Their results showed that the titanium rod experienced maximum stress at T11 for thoracic curves and at T12 for lumbar curves, specifically at the curve apex vertebrae. Vertebral stress distribution results revealed that the two surgical methods have different effects on spinal force transmission. In this study, Ponte osteotomy (M1) significantly reduced stress at T3–T5 compared to M0, while stress at T6 remained largely unchanged (Table 6). This suggests Ponte osteotomy achieves spinal correction through multi-level stress dispersion without significant stress concentration. Ponte osteotomy selectively releases facet joints, allowing gradual correction at multiple segments and avoiding excessive stress on a single vertebra. Conversely, PSO osteotomy (M2) substantially reduced stress in the osteotomized segments (T4–T5), but stress significantly increased distally at T7–T8 (Table 6). This finding suggests PSO may cause distal stress concentration due to changes in spinal force transmission. Mechanistically, PSO corrects spinal curvature via vertebral osteotomy, abruptly altering spinal alignment and increasing compensatory load on distal vertebrae. This likely explains the biomechanical basis for the increased clinical risk of adjacent segment degeneration after PSO (23). Therefore, in patients with long-segment kyphotic deformity, clinicians should carefully assess distal vertebral conditions after PSO, potentially extending the fixation segments to distribute stress effectively.

PSO was first reported in 1985. It involves extensive surgical resection across three spinal columns, preserving only the anterior longitudinal ligament. Currently, it is indicated for kyphotic deformities below 40° (24). Zhao et al. (25) reported 11 cases of rod breakage among 123 AS patients with thoracolumbar kyphosis treated by PSO, with an incidence rate of 8.9%. Stress and displacement outcomes of the internal fixation system further highlight biomechanical differences between the two surgical techniques. Internal fixation stress in the Ponte osteotomy group was significantly lower than in the PSO osteotomy group, while displacement was greater. This finding is directly associated with the distinct mechanical correction mechanisms of the two surgeries. PSO requires substantial distraction-compression forces via the internal fixation system to maintain wedge closure after vertebral osteotomy. This results in higher axial and bending loads on fixation devices, increasing the risk of rod breakage and screw loosening. These findings align with previous studies indicating a high rate of mechanical complications following PSO. Conversely, Ponte osteotomy achieves gradual correction by releasing the facet joints. Internal fixation primarily provides stabilization rather than active correction, leading to lower stress and a reduced risk of internal fixation failure. In this study, internal fixation displacement in the PSO group was smaller than that in the Ponte group (0.623 < 0.774, 0.339 < 0.435, 0.524 < 0.667, 0.471 < 0.586, 0.619 < 0.773, and 0.388 < 0.516). Because of small differences in resection angles, displacement differences were not substantial. Additionally, the moderate ROM preserved by Ponte osteotomy slightly increases internal fixation displacement. However, this displacement is within a physiologically acceptable range and does not compromise overall stability. Instead, it may reduce internal fixation fatigue fractures associated with overly rigid fixation.

This study has several limitations: ① It involved finite element analysis of a single case. Thus, model parameters may not fully reflect individual differences. ② The model did not account for dynamic factors such as muscle forces, and ligaments were simulated as linear elastic materials rather than incorporating their nonlinear, hyperelastic properties. These simplifications, while common in comparative FE studies, likely result in an overestimation of segmental motion and an underestimation of overall spinal stiffness. Consequently, the absolute values of vertebral stress and internal fixation stress might be influenced. However, since these simplifications were applied consistently across all models (M0, M1, M2), the core comparative conclusions regarding the biomechanical differences between Ponte and PSO osteotomies are expected to remain valid. ③ During model construction, other factors such as material homogeneity, mesh division, and boundary conditions were simplified, which might also cause deviations between the model and the actual anatomical structures (26). Future studies could use a variety of patient-specific models or parametric analysis methods to verify the universality of the results in the population to validate the long-term mechanical stability of both surgical methods across varying deformity severities.

In conclusion, Ponte osteotomy and PSO osteotomy each have distinct biomechanical advantages. Ponte osteotomy distributes stress effectively and involves low-load fixation, making it suitable for patients with moderate deformities or high surgical risks. PSO osteotomy offers strong correction capability and high stability, making it preferable for sagittal reconstruction of severe deformities. These findings provide critical biomechanical evidence for individualized surgical procedure selection, helping to minimize postoperative complications and enhance long-term outcomes.

## Data Availability

The raw data supporting the conclusions of this article will be made available by the authors, without undue reservation.
